# New methodology to process shifted excitation Raman difference spectroscopy data: a case study of pollen classification

**DOI:** 10.1038/s41598-020-67897-4

**Published:** 2020-07-08

**Authors:** F. Korinth, A. S. Mondol, C. Stiebing, I. W. Schie, C. Krafft, J. Popp

**Affiliations:** 1 Leibniz Institute of Photonic Technology, Albert-Einstein-Straße 9, 07745 Jena, Germany; 2 Department of Medical Engineering and Biotechnology, University of Applied Sciences, Carl-Zeiss-Promenade 2, 07745 Jena, Germany; 3 Institute of Physical Chemistry and Abbe Center of Photonics, Friedrich Schiller University Jena, Helmholtzweg 4, 07743 Jena, Germany

**Keywords:** Plant sciences, Chemistry

## Abstract

Shifted excitation Raman difference spectroscopy (SERDS) is a background correction method for Raman spectroscopy. Here, the difference spectra were directly used as input for SERDS-based classification after an optimization procedure to correct for photobleaching of the autofluorescence. Further processing included a principal component analysis to compensate for the reduced signal to noise ratio of the difference spectra and subsequent classification by linear discriminant analysis. As a case study 6,028 Raman spectra of single pollen originating from plants of eight different genera and four different growth habits were automatically recorded at excitation wavelengths 784 and 786 nm using a high-throughput screening Raman system. Different pollen were distinguished according to their growth habit, i.e. tree versus non-tree with an accuracy of 95.9%. Furthermore, all pollen were separated according to their genus, providing also insight into similarities based on their families. Classification results were compared using spectra reconstructed from the differences and raw spectra after state-of-art baseline correction as input. Similar sensitivities, specificities, accuracies and precisions were found for all spectra with moderately background. Advantages of SERDS are expected in scenarios where Raman spectra are affected by variations due to detector etaloning, ambient light, and high background.

## Introduction

Raman spectroscopy is a vibrational spectroscopy technique that is used for the assessment of the chemical composition of samples. Even complex biological samples can be analyzed in a non-destructive and label-free manner and classified using their specific molecular fingerprints assessed by this method^1–4^. However, intense and strongly varying backgrounds, e.g. due to autofluorescence (with or without photobleaching), detector etaloning effects and ambient light, are an often occurring challenge in Raman spectroscopy. If the Raman intensity is too low relative to the background intensity, Raman bands are hard to discern or are masked completely. Although it cannot be excluded that an autofluorescence background contains useful information, background correction procedures are state-of-art in Raman spectroscopy of biological material. Autofluoerescence contributions in Raman spectra seem to be sensitive, but specificity might be problematic due to bleaching and quenching effects, which are prone to variations and lack proper reproducibility.

Therefore, different approaches were suggested to tackle the challenge of intense and varying backgrounds. One option for autofluorescence is the destruction of the fluorophores by photobleaching^5–7^, which is rather time-consuming and might cause side effects, such as sample contamination with chemiphotobleaching agents or thermal stress due to extended laser exposure of the sample. A review divided other techniques roughly into two groups: computational and instrumental background correction methods^8^. Examples of typically used computational background correction algorithms are extended multiplicative signal correction^9,10^ (EMSC), multiplicative signal correction^11^ (MSC), rubberband^12,13^, sensitive nonlinear iterative peak^14^ (SNIP), and polynomial fittings^15^. These approaches often require high computational effort and need experienced personal for the data analysis. Cordero et al. corrected a high fluorescence background in Raman spectra of bladder biopsies using EMSC^16^. For in vivo Raman spectra of colorectal tissue Bergholt et al. corrected autofluorescence background by a high-order polynomial fitting^17^. The in vivo acquired Raman spectra of brain cancer by Desroches et al. were also background corrected using a polynomial^18^. Galli et al. found a high fluorescence background in Raman spectra of brain biopsies, where 88.4–96.5% of the collected intensities were attributed to fluorescence. For separating the background-free Raman signal and the fluorescence profile a baseline estimation toolkit was used. The authors concluded that the classification was best, when both information were used^19^.

Instrumental background correction methods for fluorescence rejection are time-gating approaches, where the fast Raman scattering is detected before the slower fluorescence emission, and phase or wavelength modulated techniques, where the Raman scattering changes according to the wavelength or phase modulation whereas the fluorescence emission does not^8^. Another promising method is shifted excitation Raman difference spectroscopy (SERDS)^20^.

SERDS belongs to the instrumental background correction methods, which uses two slightly shifted excitation wavelengths to acquire two Raman spectra consecutively at the same lateral position. The shift in excitation wavelength is chosen to be only a few nanometers, leading to two slightly shifted Raman spectra with the same fluorescence background profile, since the same fluorophores are excited. After subtraction of the shifted Raman spectra from each other, the resulting difference spectrum is ideally free of background contributions and only contains Raman information. Furthermore, other constant spectral contributions such as ambient light or the system transfer function (e.g. detector etaloning effects) can be suppressed^21^. Proof of principle studies demonstrated SERDS using several combinations of solvents and dyes as model analytes, especially for the introduction of new lasers with two or more excitation wavelengths^20,22–25^. Sowoidnich and Kronfeldt analyzed different laser wavelengths for SERDS experiments on parts of beef and pork tissues like fat, connective tissue, bone and meat^26^. Noack et al*.* conducted SERDS measurements to measure algae cultivation samples and monitor sulfated exopolysaccharides (EPS) concentrations in the reactors. For this, 10 raw spectra were averaged, smoothed and baseline corrected before the subtraction. A principle component analysis (PCA) and different regression models were then applied to the smoothed difference spectra to determine the EPS concentration, which worked poorly for the partial least squares regression (PLSR) model, but very well for the support vector regression (SVR) model^27^. Martins et al*.* studied molar teeth ex vivo and human skin in vivo using SERDS with an excitation wavelength of 830 nm/830.5 nm and regular Raman spectroscopy at 1,064 nm as a control. For data analysis the difference spectra were integrated to reconstruct the Raman spectrum^28^. Gebrekidan et al*.* measured difference spectra of pig tissue (bone, fat, gland and mucosal). After a sophisticated data processing including normalization, first baseline correction, reconstruction and second baseline correction to receive fluorescence-free pure Raman spectra, a classification by PCA was performed^29^. By measuring a plate of clear polystyrene, Maiwald et al*.* showed that SERDS was able to filter out ambient light passing through polystyrene. They also conducted SERDS in an orchard measuring the wax on the skin of an apple and the chlorophyll in a leaf using a handheld device with a high numerical aperture^30,31^. Schmälzlin et al*.* obtained SERDS images from different samples, e.g. cross-section of a pig ear, skin and a dissolving brown sugar cube using a custom-built multi-focus probe head and an integral field spectrograph. This system was able to simultaneously detect 400 spectra delivered by the probe head of 20 × 20 pixels^32^.

Since photobleaching and intensity variations due to e.g. laser power and filter characteristics often result in varying background intensities, most difference spectra are not completely background-free. This makes additional background correction steps necessary. Also reconstruction steps are usually implemented to transform the difficult to interpret difference spectra into accustomed Raman spectra. There are several reconstruction approaches, such as deconvolution, linear data manipulation, integration, kernel function, or non-negative least squares fitting^33–34^. These reconstruction methods always harbor the risk of introducing artefacts into the reconstructed Raman spectrum, due to the correlation between the fixed wavelength shift and varying Raman band widths^35^.

As a case study pollen samples of eight different plant genera were investigated. Pollen are a valuable case study, since their Raman spectra experience intensity differences in the fluorescence backgrounds (see supplementary information in Ref.^36^). In palynology, pollen are taxonomically evaluated under a microscope considering their morphology. This is time-consuming and requires a highly trained expert to differentiate several hundreds of different pollen. There are several ideas for automatization and technical improvement of this gold standard^37–39^. Other spectroscopic approaches like infrared absorption^40–42^, laser-induced breakdown^43^ or Raman scattering have been applied^35,43–55^. Raman spectroscopy was implemented to build a spectral database of pollen including a chemometrical classification by their growth habit^36^.

The approach in this work to differentiate several pollen genera uses difference spectra, that were obtained by novel processing of SERDS data, and lends itself as a case study to classify biological samples. The new streamlined method to handle and classify SERDS data of biological samples is based on their single difference spectra without a reconstruction step to retrieve the familiar profile of Raman spectra or baseline correction procedures. Furthermore, spectra were reconstructed from the differences and Raman spectra were processed by state-of-art baseline correction. For classification using difference spectra, reconstructed spectra and baseline corrected spectra as input, a PCA followed by a linear discriminant analysis (PCA-LDA) was chosen. The classification results were compared with respect to sensitivities, specificities, accuracies and precisions.

## Results and discussion

### Data acquisition and processing

**Figure 1:**
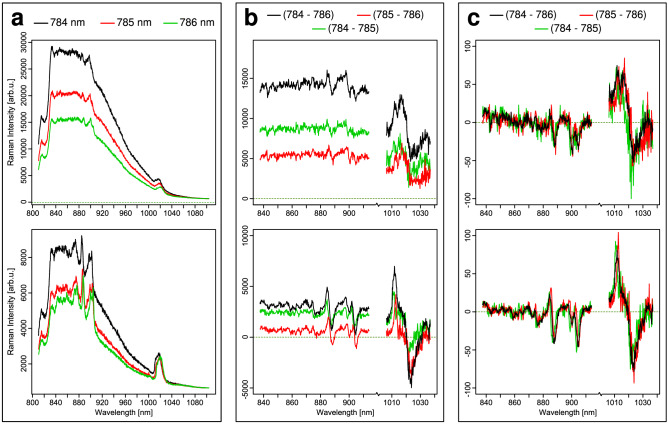
Overview of the data processing steps. top row, spectra of one birch pollen grain; bottom row, spectra of one hazel pollen grain; (**a**) raw spectra at the three different excitation wavelengths; (**b**) raw difference spectra; (**c**) normalized and optimized difference spectra; the color codes of excitation wavelengths are indicated on top.

Representative raw spectra of a single birch and hazel pollen grain at three consecutively measured excitation wavelengths of λ_1_ = 784 nm (130 mW), λ_2_ = 785 nm (180 mW) and λ_3_ = 786 nm (200 mW) are shown in Fig. 1a. The series of consecutive measurements started with 784 nm followed by 785 nm and 786 nm. The spectra show similarities in e.g. amide bands (1,310 and 1,650 cm^−1^) and the sporopollenin bands (1,007, 1,454 and 1614 cm^−1^) but vary in intensity. When separately evaluating the two sets of Raman spectra, the Raman signals shift by approx. 16 cm^−1^ per 1 nm wavelength shift. Although the laser intensities increased from 130 to 180 mW and 200 mW for 784 nm, 785 nm, and 786 nm, the signal intensities decrease in the same order. This is due to photobleaching of the autofluorescence background. Therefore, the decrease of spectral background during the onset of the measurements is stronger than the increase of the Raman bands due to elevated laser intensities. Figure 1b shows the three resulting difference spectra. For the red and green difference spectra a 1 nm shift (green: 784–785 nm; red: 785–786 nm) was realized, whereas the black difference spectrum resulted from a 2 nm shift. A significant, variable offset remained between the difference spectra, clearly originating from the varying fluorescence profile, which is maximum for the 2 nm shift. Figure 1c shows the normalized and optimized difference spectra (see data preprocessing in the methods section) of aforementioned difference spectra. The background is successfully corrected. Closer inspection reveals that a 1 nm shift results in difference spectra with higher noise than the 2 nm shift due to smaller amplitudes in the raw differences (see Fig. 1b). The shift in excitation wavelength should be near the full width at half maximum (FWHM) of the Raman band for the proper interpretation and reconstruction of a Raman spectrum. But since Raman bands have different FWHM as can be seen in Fig. 1a, there is not one wavelength shift that fits all FWHM of the Raman bands. Furthermore, technical parameters such as the transmission range of the laser line filter limit the wavelength shift to 2 nm.

Therefore, further data analysis was performed on the wavelength pair 784–786 nm. A trend is evident that negative difference bands are more intense than positive difference bands, which is a consequence of optimization step and the higher laser intensity at 786 nm. In the optimization step the subtrahend is multiplied by a factor to compensate for differences in background intensity. Since the subtracted spectra with the excitation wavelength of 786 nm always have a lower background due to photobleaching, all spectra are multiplied by an optimization factor larger than 1 resulting also in higher peak intensity and therefore more intense negative difference peaks.

**Figure 2:**
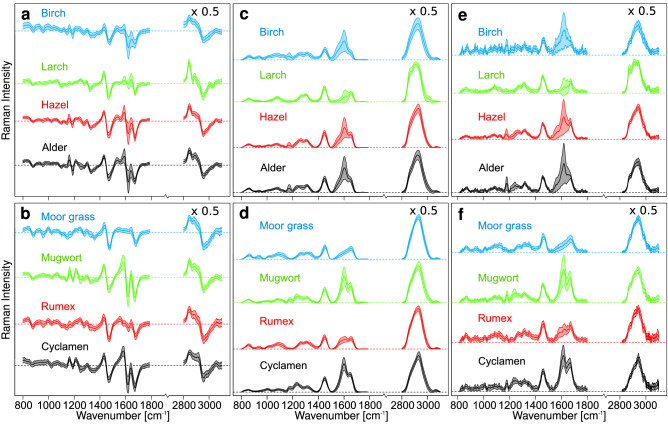
Mean and standard deviation of difference spectra (784–786 nm) after normalization and optimization for tree pollen (**a**) and non-tree pollen (**b**), spectra reconstructed from differences after SNIP baseline correction of tree pollen (**c**) and non-tree pollen (**d**) and Raman spectra (λ_ex_ = 784 nm) after SNIP baseline correction for tree pollen (**e**) and non-tree pollen (**f**). High wavenumber regions were multiplied by 0.5.

Figure 2 gives an overview of the mean spectra (dark) and their respective standard deviation (shaded). Figure 2a shows the four tree pollen difference spectra and Fig. 2b the four non-tree pollen difference spectra for the 2 nm shift, that constitute the basis for the following classification. Figure 2c, d show the reconstructed Raman spectra after baseline correction, and Fig. 2e, f the raw Raman spectra (λ_ex_ = 784 nm) after baseline correction for comparison. Spectral differences in the tree pollen occur between 1,600 and 1,750 cm^−1^, and in the high wavenumber region for larch, most likely due to higher lipid contributions of the conifer typical essential oils. Differences in non-tree pollen are also observed between 1,600 and 1,750 cm^−1^ and additionally in the low wavenumber region below 1,200 cm^−1^, which can be explained by their different families and growth habits (i.e. herb, grass, and shrub). One exception is rumex and cyclamen, which show a distinct difference in band structure although they stem from the same growth habit. In moor grass, the band near 1,600 cm^−1^ is weak. The standard variations of the 1,600 cm^−1^ band is high for all pollen. In particular, some Raman spectra of birch, larch, hazel, alder and rumex pollen also have weak intensities near 1,600 cm^−1^. The reconstruction results in spectra that properly mimic the raw Raman spectra after both data were baseline corrected using the SNIP algorithm. The main difference between reconstructed and raw spectra is that the reconstruction algorithm reduces the spectral resolution. It is important to note that the subtraction algorithm does not alter the spectral resolution of the difference spectra.

### Classification of tree vs non-tree pollen data

In the first classification step the whole data set was divided into a training data set and a test data set. A PCA was performed on the whole training data set of all pollen samples to separate major variations in the lower principal components (PCs) from noise in the higher PCs. Supplementary Fig. S1 shows the loadings of the first 15 PCs and the loading of the LDA model. Only the first 10 PCs accounting for ca. 68.6% of the variance were used for training the LDA model (for the explained variance curve see Supplementary Fig. S2). Intense variations in the loadings of the first 10 PCs can easily be seen. Almost no information is anymore provided in PC 14 and higher, whereas the high wavenumber region is dominated by noise starting from PC 11 due the lower quantum efficiency of the detector and hence lower signal intensities. A band assignment is not straightforward in the case of PC loadings based on difference spectra, since their variations are related to positive and negative Raman difference bands. This is also the case for the LD loading presented in Supplementary Fig. S1d. In the high wavenumber region the CH_3_ stretching (symmetric and asymmetric) bands, the CH_2_ stretching (symmetric and asymmetric) bands and the CH stretching bands overlap to a very broad convoluted band structure. Due to the 2 nm shift in excitation wavelength a lot of signal intensity is lost in the difference spectrum as the bands are shifted into each other. This, combined with the lower quantum efficiency of the detector, leads to a LD1 spectrum with more noise in the high wavenumber region.

**Figure 3:**
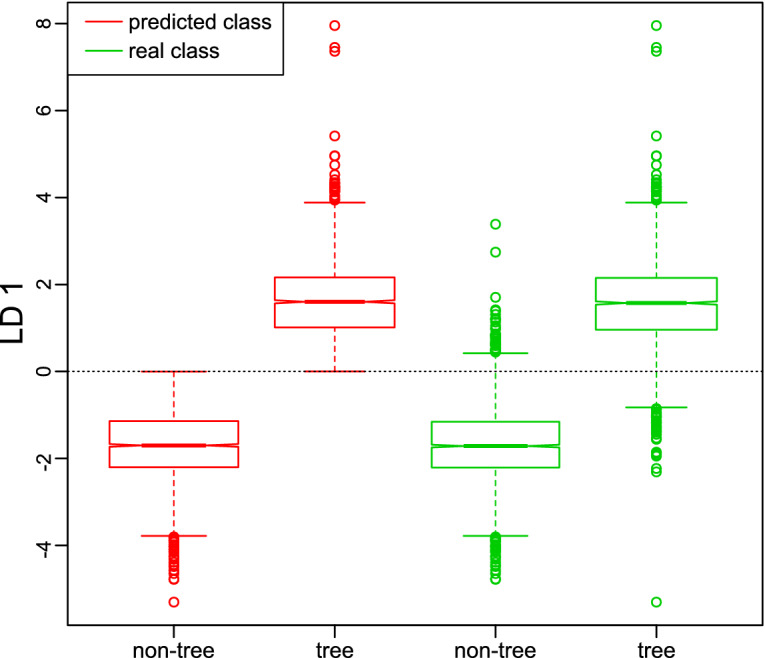
Box and whiskers LDA score plot for classification of tree vs. non-tree. Red, predicted classes of the scores of the test data set; green, real classes of the scores of the test data set.

The LD scores of the separation between tree pollen and non-tree pollen are shown in the box and whiskers plots in Fig. 3. In the prediction of the test data, all scores with a negative LD1 score belong to the non-tree class, all scores with a positive LD1 score belong to the tree class. Some misclassifications can be seen for the real classes. However, the medians and 0.25/0.75 quantiles are well separated and away from the class boundary at 0.

The confusion matrix is provided in Table 1. High sensitivity, specificity, accuracy and precision of over 95% are achieved with the constructed PCA-LDA model on average for all classifiers. The two classes can be very well separated using the normalized and optimized difference spectra and the constructed PCA-LDA model. Of the 133 misclassified tree pollen 22% were alder, 28% hazel and 50% birch pollen. Larch pollen contain a lot of lipids due to the conifer-typical essential oils, as also indicated in the difference spectra in Fig. 2, which leads to a clear separation without any misclassification.

**Table 1:** Confusion matrix of non-tree versus tree classification: real classes vs. predicted classes of the classified test spectra; sensitivity, specificity, accuracy and precision in %.

Tree vs. non-tree	Real classes	
	Non-tree	Tree
Non-tree, predicted	2,700	133
Tree, predicted	86	2,371
Sensitivity	96.9	94.7
Specificity	94.7	96.9
Accuracy	95.9	95.9
Precision	95.3	96.5

### Classification of the different pollen genera

To further analyze the feasibility of classification schemes based on SERDS difference spectra, the data set was separated into their simplified growth habits, i.e. tree and non-tree data, to classify each group into their genera. Again, the data sets were split into training and test, and a PCA-LDA model including internal cross validation was implemented for each group. The LD loadings for the discrimination of the different tree pollen types are shown in the Supplementary Fig. S3a and for the different non-tree pollen types in the Supplementary Fig. S3b. As before, the loadings and their interpretation are very complex. Nevertheless, LD3 for the non-tree separation shows a less intense spectrum. Consequently the noise has a much higher impact on the spectrum, which can be seen especially for the high wavenumber region.

**Figure 4:**
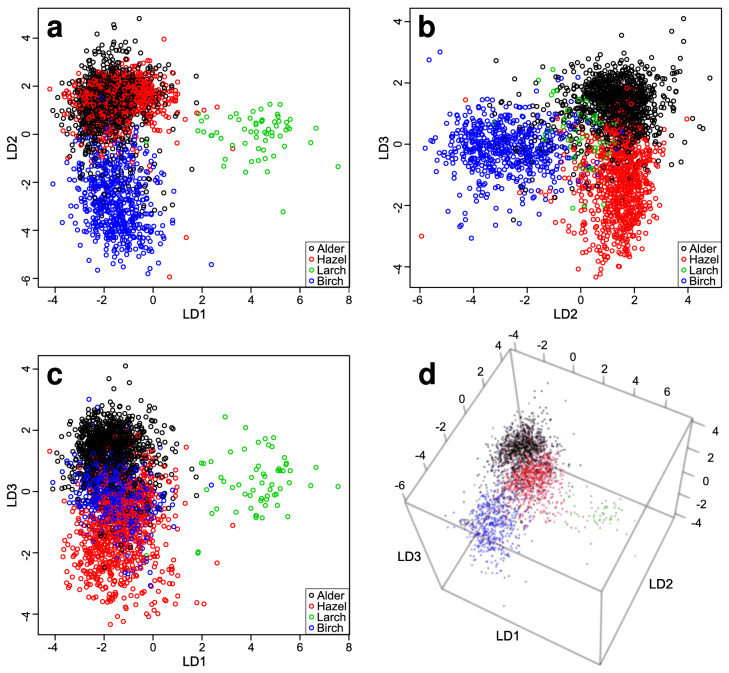
LDA score plots for classification and separation of different tree pollen genera. (**a**) LD1–LD2 plane of the score plot; (**b**) LD2–LD3 plane of the score plot; (**c**) LD1–LD3 plane of the score plot; (**d**) 3D score plot.

For LDA modelling of the different tree pollen types, the first 13 PCs were used explaining a cumulative variance of 78.6% (for the explained variance curve see Supplementary Fig. S4). The LD scores for the different tree pollen types were successfully separated, which is shown in the LD score plot (Fig. 4). LD1 separates larch from the other trees, LD2 separates birch from the other trees and LD3 grossly separates alder from hazel. The 3D score plot shows the separation of the scores of each type.

The classification results of the different tree pollen genera are summarized in Table 2. Especially the rates for the larch pollen samples show the best values: a specificity of 99.8% and an accuracy of 99.7% are the highest values for all pollen sample classifications. This is not surprising since larch is from a different family (Pinaceae) than the other three tree types (Betulaceae). Due to the high lipid content, larch pollen did not sediment very well onto the substrate causing an overall low number of automatically detected larch pollen samples for Raman spectra acquisition (in total 61 spectra). For the separation of the tree pollen types, approx. 90% of alder pollen, 85% of hazel pollen, 97% of larch pollen and 94% of birch pollen were correctly classified.

**Table 2:** Confusion matrix for classification and separation of tree pollen types.

	Real classes			
	Alder	Hazel	Larch	Birch
Alder, predicted	947	107	0	19
Hazel, predicted	54	696	2	16
Larch, predicted	3	2	59	0
Birch, predicted	52	14	0	533
Sensitivity	89.7	85.0	96.7	93.8
Specificity	91.3	95.7	99.8	96.6
Accuracy	90.6	92.2	99.7	96.0
Precision	88.3	90.6	92.2	89.0

Upper part: real classes vs. predicted classes of the classified test spectra; lower part: sensitivity, specificity, accuracy and precision in %.

**Figure 5:**
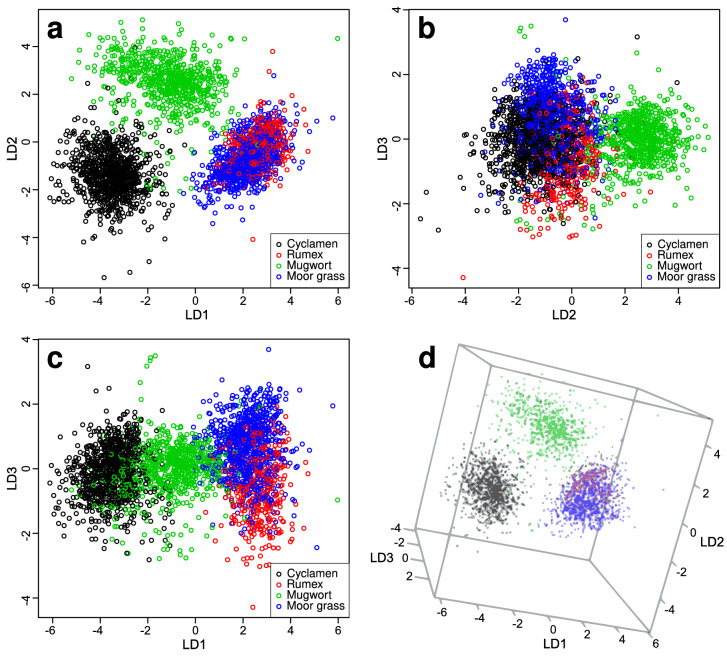
LDA score plot for classification and separation of different non-tree pollen types. (**a**) LD1–LD2 plane of the score plot; (**b**) LD2–LD3 plane of the score plot; (**c**) LD1–LD3 plane of the score plot; (**d**) 3D score plot.

For the discrimination of the non-tree data set 11 PCs were included after an internal cross validation of the training data set, explaining 61.8% of the cumulative variance (the explained variance curve see Supplementary Fig. S5). The PCA-LDA model was then used for the classification of the test data set into the four different non-tree types: mugwort, cyclamen, moor grass and rumex. The score plots of the test data classification are shown in Fig. 5. The cyclamen scores are separated from moor grass by LD1, and mugwort from all other non-tree pollen by LD2. The rumex scores can be best separated by LD3, but still exhibit a significant overlap with moor grass. In the 3D plot the scores of cyclamen and mugwort are very well separated from the other two classes, whereas rumex and moor grass have a larger overlap, thus making it hard to separate the two pollen types from one another. Table 3 shows the results of the classification of the non-tree pollen types and the corresponding classifiers in form of a confusion matrix.

**Table 3:** Confusion matrix for classification and separation of non-tree pollen types.

	Real classes			
	Cyclamen	Rumex	Mugwort	Moor grass
Cyclamen, predicted	897	0	18	0
Rumex, predicted	0	267	16	168
Mugwort, predicted	16	0	676	0
Moor grass, predicted	0	96	10	622
Sensitivity	98.2	73.6	93.9	78.7
Specificity	99.0	92.4	99.2	94.7
Accuracy	98.8	89.9	97.8	90.2
Precision	98.0	59.2	97.7	85.4

Upper part: real classes vs. predicted classes of the classified test spectra; lower part: sensitivity, specificity, accuracy and precision in %.

In the separation between the different non-tree pollen, 98% of the cyclamen pollen, 74% of the rumex pollen, 94% of the mugwort pollen and 79% of the moor grass pollen were correctly classified. Rumex and cyclamen are herbs, mugwort a shrub type plant and moor grass a grass. The SERDS spectra of rumex and moor grass pollen are quite similar resulting in the most misclassifications. The classification of grass pollen genera by Raman spectroscopy was challenging which was also found by Mondol et al.^36^.

### Comparison with classification of reconstructed and raw Raman spectra

Reconstructed and raw Raman spectra after baseline correction as shown in Fig. 2 for tree and non-tree pollen were subjected to the analogous PCA-LDA classification. Confusion matrixes are presented in Supplementary Table S2. To simplify the comparison, average values for sensitivity, specificity, accuracy and precision were calculated and displayed in Supplementary Table S1. The classification rates of tree versus non-tree pollen agreed well for difference spectra and spectra at 784 and 786 nm excitation, but are 2–3% lower for the reconstructed spectra. The classification rates of tree types varied between 88 and 98%, and the reconstructed spectra tended to give lower values than difference, 784 nm and 786 nm data. The classification rates of non-tree types show even stronger variations between 85 and 98%. Here, the 784 and 786 nm data gave slightly better results. Overall, the classification results differed only little. Another comparison of baseline corrected Raman spectra with difference spectra collected by wavelength modulation was presented for leukocytes and tumor cells and confirmed only small difference in classification rates^57^. Similar to this pollen study, the spectral background was moderate in the previous cell study. A true benefit of SERDS is expected for high spectral background and contributions from ambient light and etaloning which cannot be suppressed by state-of-art baseline correction and goes well beyond.

## Conclusions

In this contribution, the methodology was described to use difference spectra based on SERDS for classification of pollen data. Its main advantage is the analysis of single difference spectra by PCA and subsequent LDA with few PCs as input without complex data pre-processing. An optimization procedure compensated the photobleaching effects and minimized the remaining background in the difference spectra, whereas the normalization was necessary to obtain the same intensity range for all measured pollen difference spectra. This resulted in a classification based on the spectral features of the difference spectra and not based on the overall intensity of the Raman spectrum of a pollen sample. A further improvement would be rapid, serial acquisitions of Raman spectra at both wavelengths, which suppresses photobleaching effects and avoids the optimization procedure^58^. Since no reconstruction of the familiar profiles of Raman spectra is necessary, possible artefacts are not introduced into the spectra. The down side of this direct classification using SERDS spectra is the difficult spectral analysis of difference spectra and especially the resulting PC and LD loadings. The increase in noise level due to the subtraction of two spectra compared to a single Raman spectrum is compensated by PCA, which separated the spectral variations in the first PCs from the noise in the higher PCs. The possibility to classify different pollen samples using normalized and optimized difference spectra by a linear PCA-LDA model was successfully demonstrated. In Supplementary Table S1 the average values for sensitivity, specificity, accuracy and precision for all classifications are shown that achieved good to very good results using difference spectra as input. Since birch, hazel and alder all belong to the same family, in case of birch and alder even to the same subfamily, the pollen could even be separated on a genus level. For comparison, the reconstructed spectra and the raw spectra at 784 and 786 nm excitation after baseline correction were subjected to PCA-LDA classification. The classification rates only show small variations with a tendency of worse results for reconstructed spectra. This demonstrates the validity of our new approach based on difference spectra. The full potential of SERDS will become evident for Raman spectra that are affected by high autofluorescence background, ambient light or etaloning effects. Since the pollen detection, the laser focusing, the wavelength shifting and the data recording was fully automated, this streamlined method could be a robust and versatile system for the automated differentiation of different pollen into their genera.

## Methods

### Sample specifications

Pollen samples used in this study were collected by the Department of Indoor Climatology (University Hospital Jena, Germany) over the last two decades and transferred to the Leibniz Institute of Photonic Technology (Jena, Germany) for storage and research purposes. Eight different pollen genera were analyzed, which can be grouped into two classes based on their growth habit: tree and non-tree (see Supplementary Table S3).

### Set-up description and specification

The previously developed high throughput screening Raman spectroscopy platform (HTS-RS)^59^ was modified for the implementation of SERDS and the investigation of pollen grains. A tunable laser source (DLC DL pro 780, Toptica Photonics, Germany) with a tuning range from 765 to 805 nm in combination with an amplifier (BoosTA Pro, Toptica Photonics, Germany) was fiber-coupled into a microscope set-up by a multimode fiber with a 60 µm core diameter and 0.22 NA (Thorlabs, Germany). The booster enhanced the laser power of the three operating wavelengths (λ = 784 nm/785 nm/786 nm) to the system. The incoming laser beam passed through a clean-up filter (785 ± 1.5 nm; Semrock, USA) and was collimated using a 30 mm focal length lens (Thorlabs, Germany). The collimated beam was guided to the back aperture of the microscope objective (60×, NA = 1, water immersion, Nikon, Japan) via a dichroic notch filter (785 nm, bandwidth 89 nm; Semrock, USA) and a 45° tilted mirror (Thorlabs, Germany). At the end of the objective, the pollen grains were excited with an approximate focus spot diameter of 10 µm on the sample plane. The back reflected Raman signals from the samples were collected and projected to a 100 mm focal length lens (Thorlabs, Germany) while passing through the same objective lens and the notch filter, which blocked most of the Rayleigh signal. An extra notch filter operating at 785 nm ± 19 nm (Laser Components, Germany) was placed before the 100 mm collection lens ensuring maximal rejection of Rayleigh signal propagation. The 100 mm collection lens focused the Raman signal to a 100 µm, 0.22 NA multimode fiber (Thorlabs, Germany) coupling it to a spectrograph (IsoPlane160, Princeton Instruments, USA) with a 400 grooves/mm grating blazed at 750 nm. The Raman signals were projected to a charge-coupled device (CCD) (PIXIS-400BR-eXcelon; Princeton Instruments, USA) with an operating temperature of − 60 °C. A bright field channel was integrated into the set-up for the automation of particle detection, of various calibrations and for visualization purposes. A white LED source (Thorlabs, Germany) was employed to illuminate the sample from below and the light was guided to a CCD camera (DCC1645C, Thorlabs, Germany) via a long pass filter (Semrock, USA) and a 70 mm focal length lens (Thorlabs, Germany). The bright field microscopic image was used in an in-house developed pollen detection algorithm for the automated detection of single pollen grain. All the required translations were realized using two CONEX MFA-Series motor (Newport, USA) for xy plane and a MTS25-Z8 motor (Thorlabs, Germany) for z direction^36,59^.

### Sample preparation and data acquisition

Each pollen sample was suspended in 10 mL deionized water and pipetted onto a CaF_2_ cover slip fully immersed in deionized water. After sedimentation of the pollen, single pollen samples were automatically detected, the signal focused and measured at three different excitation wavelengths (λ_1_ = 784 nm/130 mW; λ_2_ = 785 nm/180 mW; λ_3_ = 786 nm/200 mW) before moving to the next pollen. The acquisition time for each spectrum was 0.5 s with a short dwell time of 0.5 s after each measurement to allow for wavelength shifting.

### Data preprocessing

The collected data was analyzed in R^60^ using the following packages: hyperSpec^61^, cbmodels^62^, Ramancal^63^, rgl^64^, pracma^65^, gtools^66^ and ROCR^67^. The raw spectra were first corrected for cosmic spikes^68^, wavelength calibrated in relation to λ_ex_ = 785 nm using 4-Acetaminophenole and then intensity calibrated using a white-light source calibration lamp (Raman Calibration Accessory—HCA, Kaiser Optical Systems, Inc., USA). Since the background between the different excitation wavelengths varies due to photobleaching and differences in laser intensity, the spectra of different excitation wavelengths were difference-optimized before subtraction as described previously^35^. For comparison, Raman spectra were reconstructed from the SERDS spectra by summation of the signal intensities ($S\left(n\right)= \sum_{x=1}^{n}S(x)$, where $S(n)$ corresponds to the signal intensity at pixel n) and subsequent baseline correction using the SNIP algorithm^14,69^. Furthermore, Raman spectra (λ_ex_ = 784 nm and λ_ex_ = 786 nm) were also baseline corrected (SNIP) after cosmic spike correction, wavelength and intensity calibration.

For each pollen type a Pearson correlation of the optimized difference spectra to the respective pollen type’s mean optimized difference spectrum was performed as a spectral quality control to detect outliers. To make sure that also pollen debris spectra, out-of-focus pollen spectra and pure water spectra are excluded from the data set, all spectra with a Pearson correlation coefficient below 0.52 were discarded, leading to 4–24% of spectra being discarded. However, for the larch pollen the highest percentage of 52% was discarded because of poor sedimentation and therefore a lot of out-of-focus measurements due to the high lipid content of the pollen.

The overall intensity of the Raman signal varies between pollen types. To make sure the classification occurs due to spectral features and not overall intensity differences, the difference spectra were normalized. The performed normalization was a Euclidean-distance-like area normalization.

### Data classification

The preprocessed, optimized and normalized data was separated into a test and training data set. The training data set was chosen in a way that every pollen type was represented by the same number of spectra (ca. 60% of the smallest data set resulting in 90 spectra). The remaining spectra were used for testing. As a classification scheme the training data was first dimensionally reduced using a Principal Component Analysis (PCA) and then used to construct a Linear Discriminant Analysis (LDA) classification model. The models were internally validated using a tenfold cross validation to determine the optimal number of principal components (PCs) for building robust PCA-LDA models. In a first step 10 PCs of all eight pollen genera were classified into the two growth habits: tree and non-tree. In a second step 13 PCs of all measured tree pollen were classified based on their genera: alder, birch, hazel and larch. In the final step 11 PCs of all measured non-tree pollen were classified into their different genera: mugwort, cyclamen, moor grass and rumex. Herein, we present the Δλ_ex_ = 2 nm shift in excitation wavelength (λ_1_ = 784 nm; λ_2_ = 786 nm; Δλ_ex_ = λ_1_ − λ_2_ = 2 nm). For comparison, the same classification schemes were used on the reconstructed Raman spectra and the baseline corrected Raman spectra.

## Supplementary information

Supplementary file1 (DOCX 1166 kb)
