# Supplementary material for: New methodology to process shifted excitation Raman difference spectroscopy data: a case study of pollen classification
Source: Sci Rep. 2020 Jul 8;10:11215. doi: 10.1038/s41598-020-67897-4 (PMC7343813; doi:10.1038/s41598-020-67897-4)

**New Methodology to Process Shifted Excitation Raman Difference Spectroscopy Data: A Case Study of Pollen Classification**

F. Korinth^1^, A.S. Mondol^1^, C. Stiebing^1^, I.W. Schie^1, 2^, C. Krafft^1*^, J. Popp^1, 3^

^1^ Leibniz Institute of Photonic Technology, Jena, Albert-Einstein-Straße 9, 07745 Jena, Germany

^2^ Department of Medical Engineering and Biotechnology, University of Applied Sciences, Jena,
Carl-Zeiss-Promenade 2, 07745 Jena, Germany

^3^ Institute of Physical Chemistry and Abbe Center of Photonics, Friedrich Schiller University Jena,
Jena, Helmholtzweg 4, 07743 Jena, Germany

^*^ christoph.krafft@leibniz-ipht.de

**Supplementary Information: Tables**

**Supplementary Tables S1. Average sensitivity, specificity, accuracy and precision in % using difference, reconstructed, 784 nm and 786 nm excited Raman spectra as input.**

**A: tree versus non-tree classification**

| **Classifiers** | **Difference** | **Reconstructed** | **784 nm** | **786 nm** |
| --- | --- | --- | --- | --- |
| Sensitivity | 95.8 | 92.8 | 95.7 | 95.2 |
| Specificity | 95.8 | 92.8 | 95.7 | 95.2 |
| Accuracy | 95.9 | 92.7 | 95.7 | 95.1 |
| Precision | 95.9 | 92.7 | 95.6 | 95.1 |

**B: tree types**

| **Classifiers** | **Difference** | **Reconstructed** | **784 nm** | **786 nm** |
| --- | --- | --- | --- | --- |
| Sensitivity | 91.3 | 89.9 | 93.8 | 95.6 |
| Specificity | 95.9 | 95.2 | 96.8 | 98.2 |
| Accuracy | 94.6 | 93.7 | 95.9 | 97.5 |
| Precision | 90.0 | 88.3 | 91.5 | 93.6 |

**C: non-tree types**

| **Classifiers** | **Difference** | **Reconstructed** | **784 nm** | **786 nm** |
| --- | --- | --- | --- | --- |
| Sensitivity | 86.1 | 89.8 | 91.5 | 90.3 |
| Specificity | 96.3 | 97.3 | 97.7 | 97.3 |
| Accuracy | 94.2 | 95.8 | 96.4 | 95.7 |
| Precision | 85.1 | 89.3 | 90.6 | 88.8 |

**Supplementary Tables S2A: tree versus non-tree classification**

| *Reconstructed* | | **REAL CLASSES** | |
| --- | --- | --- | --- |
|  |  | *Non-tree* | *Tree* |
| **PREDICTED CLASSES** | *Non-tree* | 2579 | 179 |
|  | *Tree* | 207 | 2325 |
| Sensitivity | | 92.6 | 92.9 |
| Specificity | | 92.9 | 92.6 |
| Accuracy | | 92.7 | 92.7 |
| Precision | | 93.5 | 91.8 |

| *784 nm* | | **REAL CLASSES** | |
| --- | --- | --- | --- |
|  |  | *Non-tree* | *Tree* |
| **PREDICTED CLASSES** | *Non-tree* | 2649 | 93 |
|  | *Tree* | 137 | 2411 |
| Sensitivity | | 95.1 | 96.3 |
| Specificity | | 96.3 | 95.1 |
| Accuracy | | 95.7 | 95.7 |
| Precision | | 96.6 | 94.6 |

| *786 nm* | | **REAL CLASSES** | |
| --- | --- | --- | --- |
|  |  | *Non-tree* | *Tree* |
| **PREDICTED CLASSES** | *Non-tree* | 2607 | 79 |
|  | *Tree* | 179 | 2425 |
| Sensitivity | | 93.6 | 96.8 |
| Specificity | | 96.8 | 93.6 |
| Accuracy | | 95.1 | 95.1 |
| Precision | | 97.1 | 93.1 |

**Supplementary Tables S2B: classification of tree types**

| *Reconstructed* | | **REAL CLASSES** | | | |
| --- | --- | --- | --- | --- | --- |
|  |  | *Alder* | *Hazel* | *Larch* | *Birch* |
| **PREDICTED CLASSES** | *Alder* | 903 | 93 | 0 | 38 |
|  | *Hazel* | 83 | 703 | 2 | 8 |
|  | *Larch* | 0 | 4 | 59 | 1 |
|  | *Birch* | 70 | 19 | 0 | 521 |
| Sensitivity | | 85.5 | 85.8 | 96.7 | 91.7 |
| Specificity | | 91.0 | 94.5 | 99.8 | 95.4 |
| Accuracy | | 88.7 | 91.7 | 99.7 | 94.6 |
| Precision | | 87.3 | 88.3 | 92.2 | 85.4 |

| *784 nm* | | **REAL CLASSES** | | | |
| --- | --- | --- | --- | --- | --- |
|  |  | *Alder* | *Hazel* | *Larch* | *Birch* |
| **PREDICTED CLASSES** | *Alder* | 954 | 54 | 0 | 47 |
|  | *Hazel* | 57 | 764 | 0 | 0 |
|  | *Larch* | 5 | 2 | 61 | 1 |
|  | *Birch* | 40 | 0 | 0 | 520 |
| Sensitivity | | 90.3 | 93.3 | 100.0 | 91.5 |
| Specificity | | 93.0 | 96.6 | 99.7 | 97.9 |
| Accuracy | | 91.9 | 95.5 | 99.7 | 96.5 |
| Precision | | 90.4 | 93.1 | 89.7 | 92.9 |

| *786 nm* | | **REAL CLASSES** | | | |
| --- | --- | --- | --- | --- | --- |
|  |  | *Alder* | *Hazel* | *Larch* | *Birch* |
| **PREDICTED CLASSES** | *Alder* | 987 | 29 | 0 | 17 |
|  | *Hazel* | 29 | 788 | 2 | 5 |
|  | *Larch* | 5 | 2 | 59 | 0 |
|  | *Birch* | 35 | 0 | 0 | 546 |
| Sensitivity | | 93.5 | 96.2 | 96.7 | 96.1 |
| Specificity | | 96.8 | 97.9 | 99.7 | 98.2 |
| Accuracy | | 95.4 | 97.3 | 99.6 | 97.7 |
| Precision | | 95.5 | 95.6 | 89.4 | 94.0 |

**Supplementary Tables S2C: classification of non-tree types**

| *Reconstructed* | | **REAL CLASSES** | | | |
| --- | --- | --- | --- | --- | --- |
|  |  | *Cyclamen* | *Rumex* | *Mugwort* | *Moor grass* |
| **PREDICTED CLASSES** | *Cyclamen* | 896 | 0 | 24 | 0 |
|  | *Rumex* | 1 | 289 | 10 | 93 |
|  | *Mugwort* | 15 | 0 | 671 | 0 |
|  | *Moor grass* | 1 | 74 | 15 | 697 |
| Sensitivity | | 98.1 | 79.6 | 93.2 | 88.2 |
| Specificity | | 98.7 | 95.7 | 99.3 | 95.5 |
| Accuracy | | 98.5 | 93.6 | 97.7 | 93.4 |
| Precision | | 97.4 | 73.5 | 97.8 | 88.6 |

| *784 nm* | | **REAL CLASSES** | | | |
| --- | --- | --- | --- | --- | --- |
|  |  | *Cyclamen* | *Rumex* | *Mugwort* | *Moor grass* |
| **PREDICTED CLASSES** | *Cyclamen* | 899 | 0 | 18 | 1 |
|  | *Rumex* | 2 | 309 | 6 | 93 |
|  | *Mugwort* | 10 | 0 | 679 | 0 |
|  | *Moor grass* | 2 | 54 | 17 | 696 |
| Sensitivity | | 98.5 | 85.1 | 94.3 | 88.1 |
| Specificity | | 99.0 | 95.8 | 99.5 | 96.3 |
| Accuracy | | 98.8 | 94.4 | 98.2 | 94.0 |
| Precision | | 97.9 | 75.4 | 98.5 | 90.5 |

| *786 nm* | | **REAL CLASSES** | | | |
| --- | --- | --- | --- | --- | --- |
|  |  | *Cyclamen* | *Rumex* | *Mugwort* | *Moor grass* |
| **PREDICTED CLASSES** | *Cyclamen* | 901 | 0 | 7 | 0 |
|  | *Rumex* | 3 | 307 | 12 | 135 |
|  | *Mugwort* | 9 | 0 | 684 | 0 |
|  | *Moor grass* | 0 | 56 | 17 | 655 |
| Sensitivity | | 98.7 | 84.6 | 95.0 | 82.9 |
| Specificity | | 99.6 | 93.8 | 99.6 | 96.3 |
| Accuracy | | 99.3 | 92.6 | 98.4 | 92.5 |
| Precision | | 99.2 | 67.2 | 98.7 | 90.0 |

**Supplementary Table S3. Data Set Overview:** Name, growth habit, family, genus and number of spectra for the training and test data set of the analyzed pollen samples.

| **Name** | **Growth habit** | **Family** | **Genus** | **Training set**  **Number of spectra** | **Test set**  **Number of spectra** |
| --- | --- | --- | --- | --- | --- |
| Alder | Tree | Betulaceae | Alnus | 90 | 1056 |
| Birch | Tree | Betulaceae | Betula | 90 | 586 |
| Hazel | Tree | Betulaceae | Corylus | 90 | 819 |
| Larch | Tree | Pinaceae | Larix | 90 | 61 |
| Cyclamen | Non-tree (herb) | Primulaceae | Cyclamen | 90 | 913 |
| Rumex | Non-tree (herb) | Polygonaceae | Rumex | 90 | 363 |
| Mugwort | Non-tree (shrub) | Asteraceae | Artemisia | 90 | 720 |
| Moor grass | Non-tree (grass) | Poaceae | Molinia | 90 | 790 |

**Supplementary Information: Figures**

**
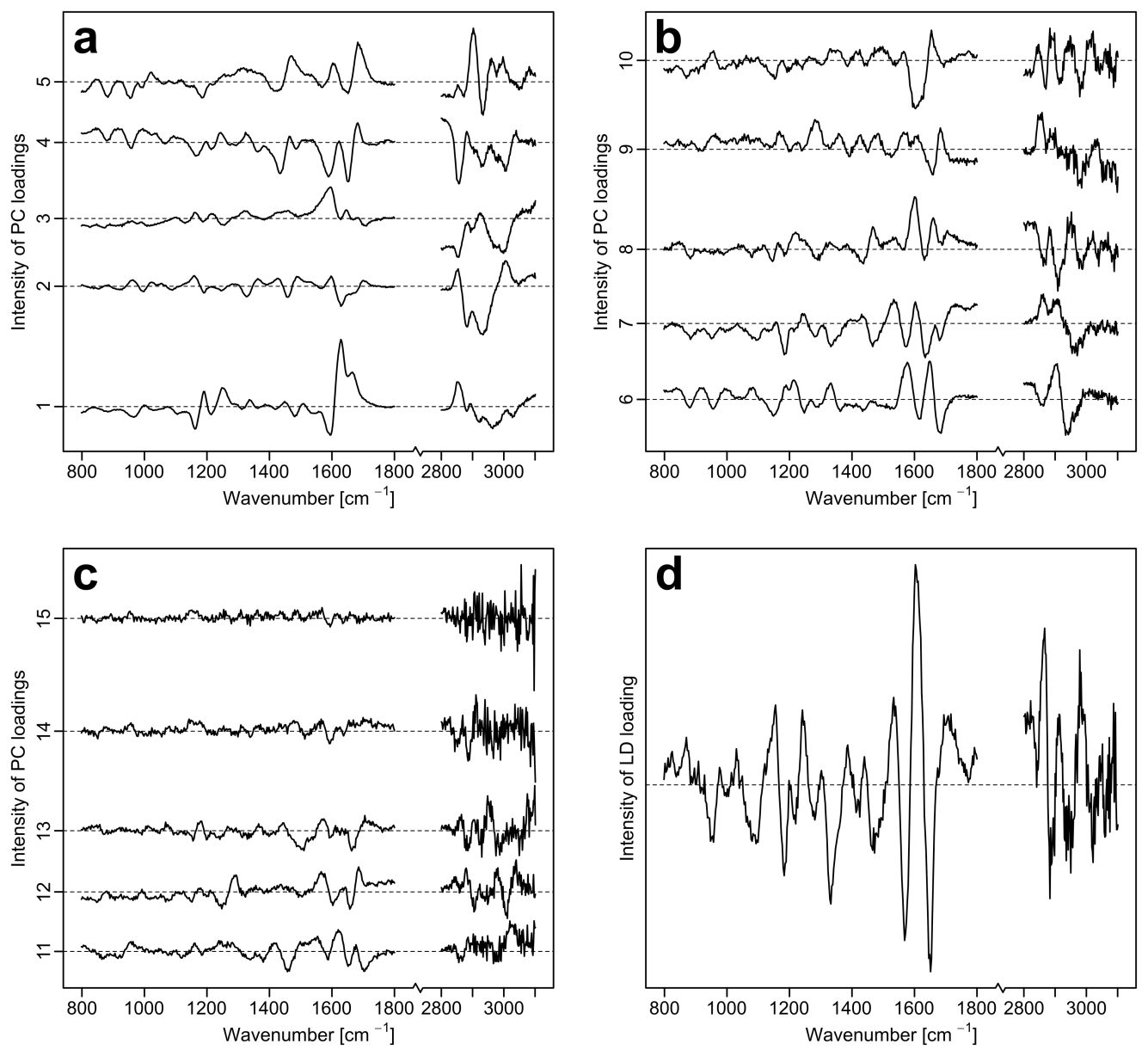
**

**Supplementary Figure S1. Loadings of the PCA and the LDA of the whole training data set (8 pollen types) – classification of tree vs. non**-**tree:** loadings 1 – 5 (a), loadings 6 – 10 (b), loadings 11 – 15 (c) of the PCA, **d**, loading of the LDA (d).


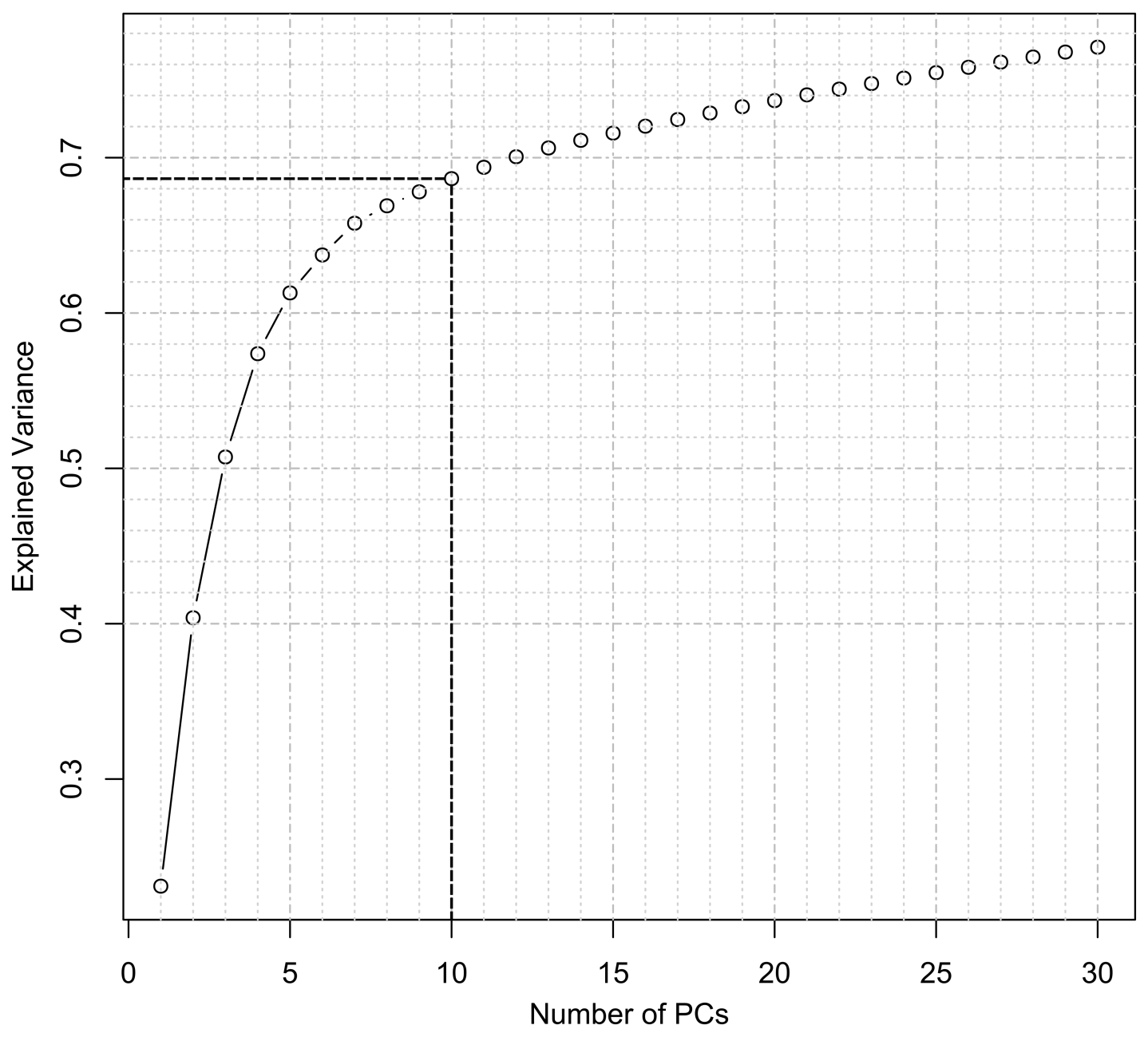


**Supplementary Figure S2. Explained variance curve of the PCA of the whole training data set (8 different pollen types) – classification of tree vs. non**-**tree,** and optimal number of PCs according to the 10-fold cross validation results (dashed line).


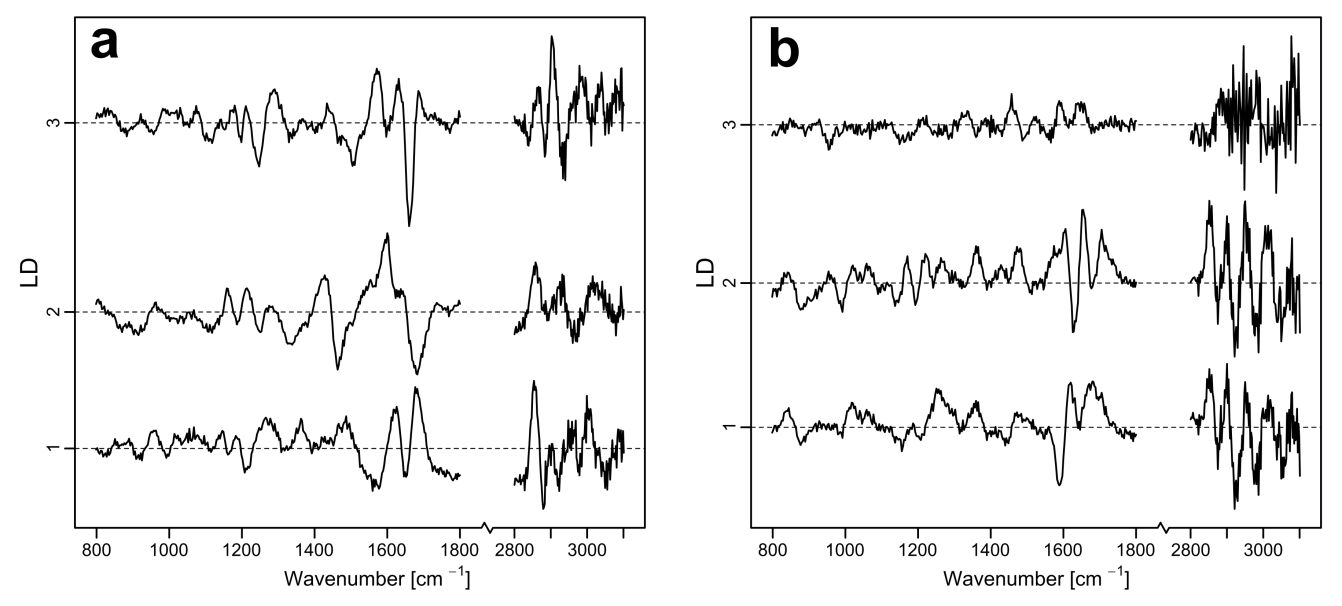


**Supplementary Figure S3. LD loadings for classification and separation of pollen genera:**

tree pollen types (A) and non-tree pollen types (B).


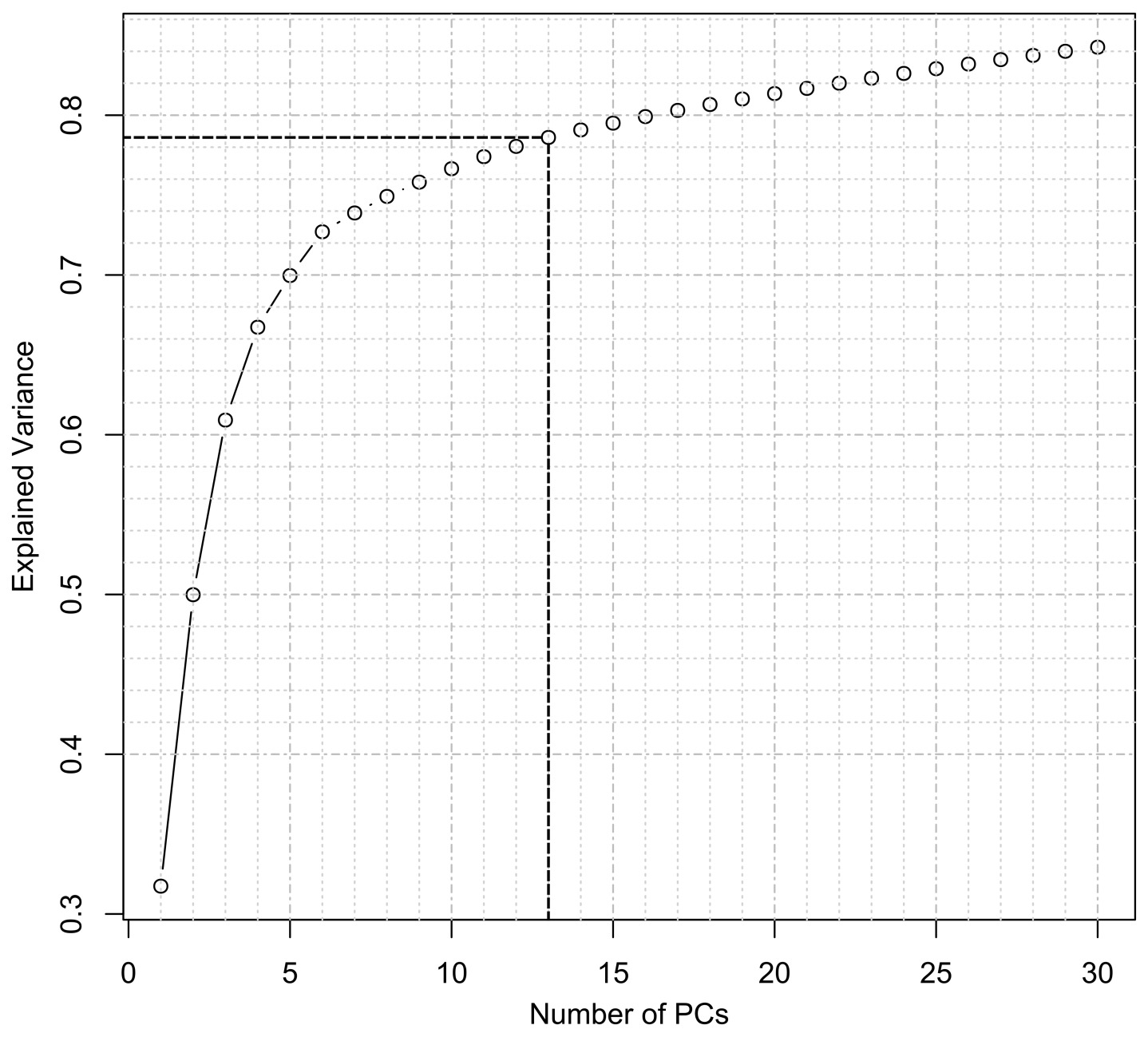
**Supplementary Figure S4. Explained variance curve of the PCA for the separation of tree genera:**

optimal number of PCs according to the 10-fold cross validation results was 13 (dashed line).

**Supplementary Figure S5. Explained variance curve of the PCA for the separation of non-tree genera:**
optimal number of PCs according to the 10-fold cross validation results was 11 (dashed line).
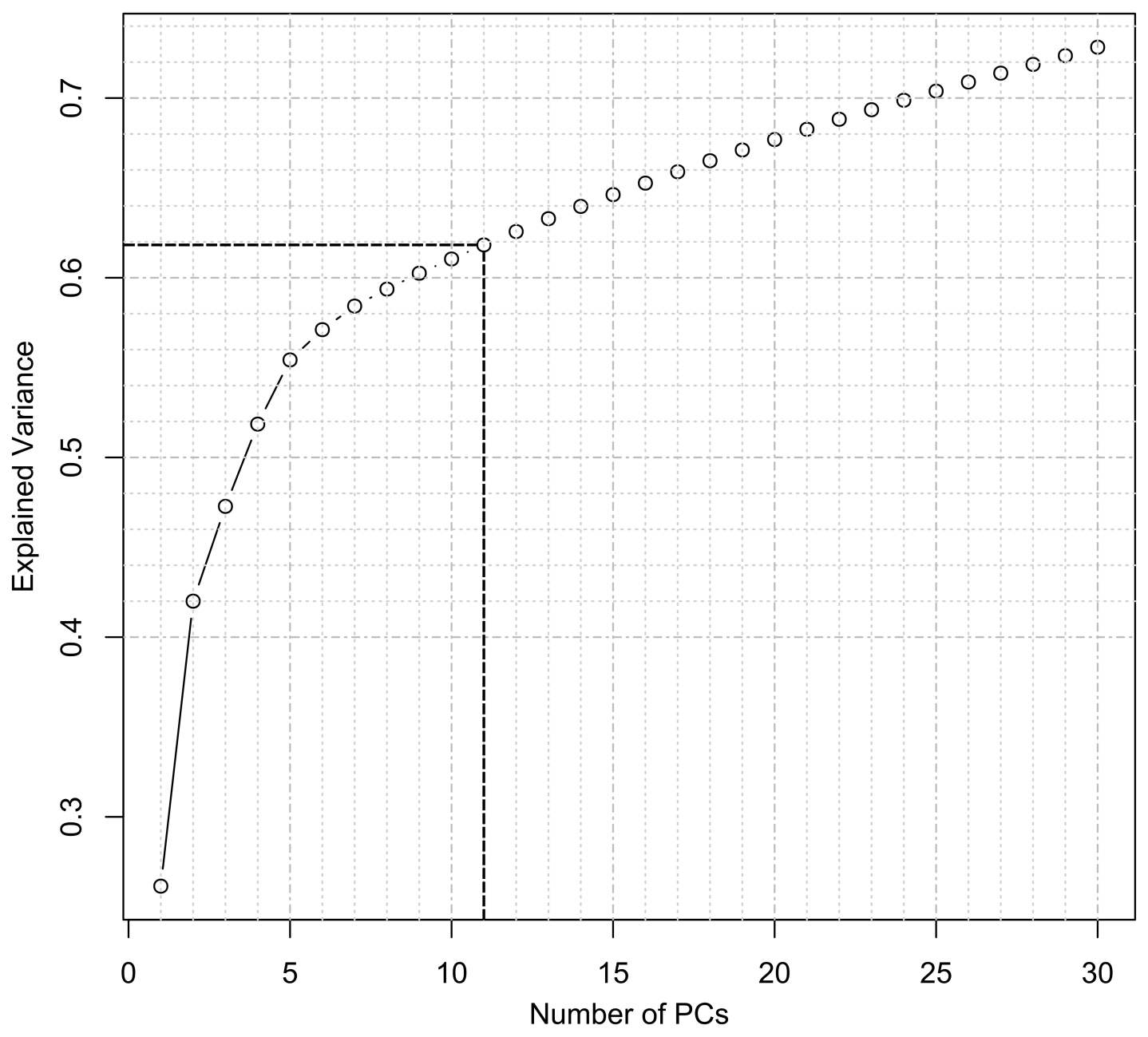

Supplement: Supplementary file 1 — Supplementary file1 (DOCX 1166 kb) [file 41598_2020_67897_MOESM1_ESM.docx]
